# Idiopathic Juvenile Osteoporosis Diagnosed in Adulthood: The First Documented Case in Georgia

**DOI:** 10.7759/cureus.83707

**Published:** 2025-05-08

**Authors:** Giorgi Akhvlediani, Nana Nakaidze, Elene Dzodzuashvili, Nino Gabidzashvili, Elene Dopidze, Ana Mikaberidze, Kristine Ambriashvili

**Affiliations:** 1 Pulmonary and Critical Care Medicine, Tbilisi State Medical University, Tbilisi, GEO; 2 Biomedical Sciences, Georgian American University, Tbilisi, GEO; 3 Endocrinology, Diabetes and Metabolism, American Hospital Tbilisi, Tbilisi, GEO; 4 Internal Medicine, Vian Hospitals, Tbilisi, GEO

**Keywords:** a case report, bone density, idiopathic juvenile osteoporosis, markers of bone metabolism, metabolic endocrinology, parathyroid hormone (pth)

## Abstract

Idiopathic juvenile osteoporosis (IJO) is a rare metabolic bone disorder characterized by bone fragility in otherwise healthy children and adolescents, with typical onset before puberty. To our knowledge, this represents the first documented case of IJO in Georgia, with a delayed diagnosis in adulthood despite a clinical history suggestive of earlier onset. A 24-year-old male with a history of childhood nephrolithiasis and intermittent vitamin D deficiency presented with progressive bone pain, joint crepitus, and worsening mobility. Imaging revealed severe osteopenia and osteoporosis, prompting an extensive metabolic and endocrine evaluation. Laboratory findings were largely unremarkable aside from episodic hypercalciuria, normal parathyroid hormone levels, and fluctuating vitamin D levels. Major secondary causes, including malignancy, hyperparathyroidism, thyroid dysfunction, chronic inflammatory disease, and malabsorption syndromes, were ruled out. A diagnosis of IJO was made by exclusion. The patient was started on calcium and vitamin D supplementation with close monitoring. This case highlights the diagnostic challenges of IJO when presentation extends into adulthood and underscores the importance of considering this condition in young adults with unexplained bone fragility, particularly in regions where it remains undocumented.

## Introduction

Idiopathic juvenile osteoporosis (IJO) is a rare primary bone disorder characterized by progressive bone demineralization, increased fracture risk, and skeletal pain in otherwise healthy children and adolescents [1,2]. Unlike secondary osteoporosis, which results from identifiable metabolic, endocrine, inflammatory, or systemic conditions, IJO is a diagnosis of exclusion, typically made after an extensive workup fails to uncover a specific cause [1-3]. The disorder most commonly presents between ages five and 15, often with bone pain, gait disturbances, and vertebral compression fractures, though severity and progression vary [1-4].

The global incidence of IJO remains unknown, with fewer than 200 cases documented in the literature, and limited genetic understanding further complicates early detection [1,2]. Due to its rarity, nonspecific clinical presentation, and lack of definitive biomarkers, IJO is frequently underdiagnosed or misdiagnosed, especially in regions with limited access to diagnostic resources [3,5]. Some cases resolve spontaneously with skeletal maturity, while others may progress to chronic disability, growth delay, or persistent bone fragility [5,6].

This case is notable for the diagnosis of IJO in a young adult, years after the typical window of onset. The delay illustrates a broader clinical challenge: recognizing IJO outside of childhood and differentiating it from secondary causes such as malignancy, hyperparathyroidism, thyroid disorders, malabsorption syndromes, and chronic inflammatory diseases. We present the first documented case of IJO in Georgia, contributing to the limited regional data and highlighting the need for greater awareness, earlier recognition, and systematic evaluation to avoid long-term complications.

## Case presentation

**Figure 1:**
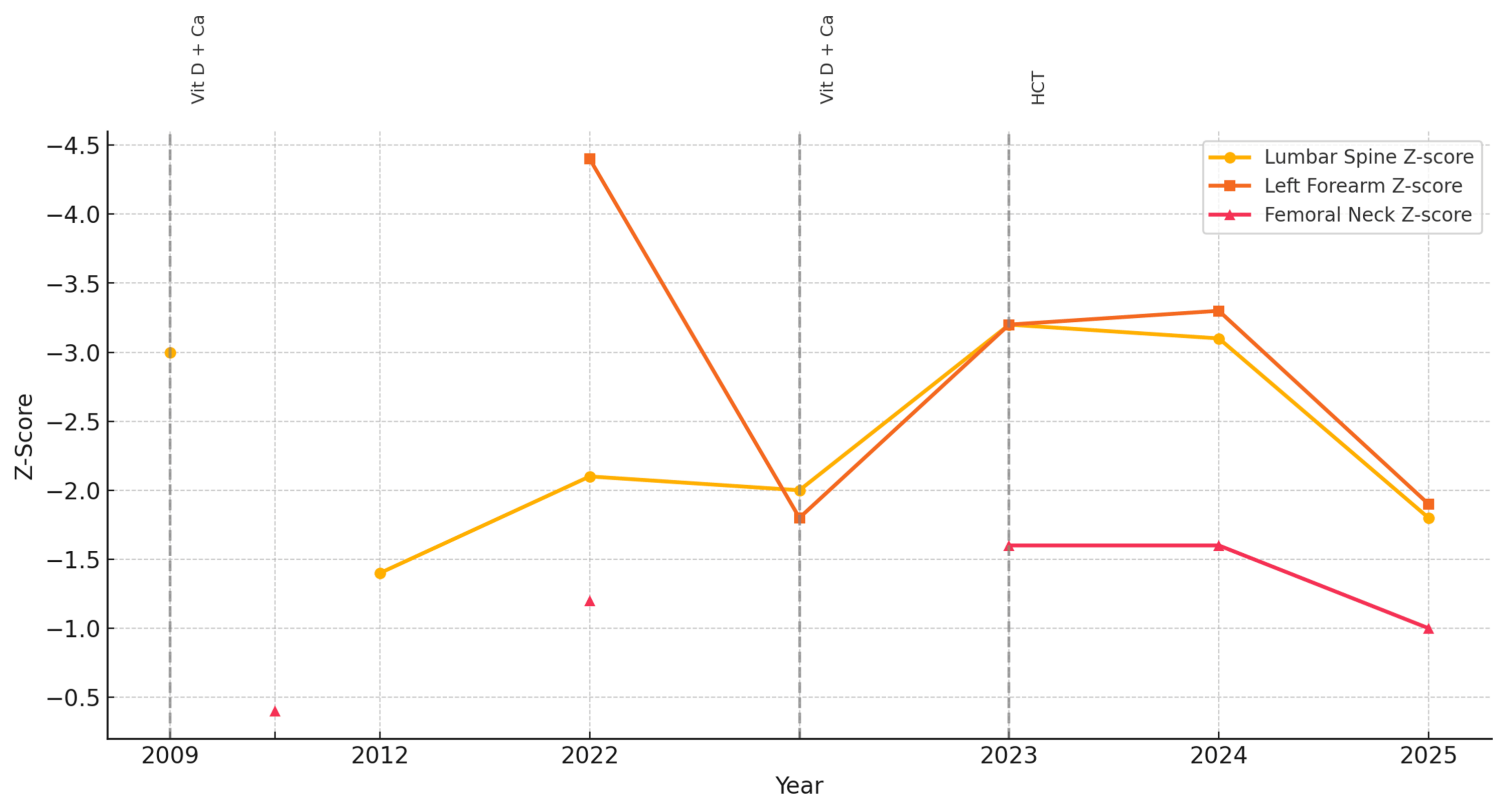
Z-score trends by site on serial DEXA scans (2009-2025) This graph illustrates serial Z-score measurements from DEXA scans at three anatomical sites from 2009 to 2025. Vertical dashed lines represent the initiation of treatment interventions: Vit D + Ca: introduction of vitamin D and calcium supplementation; HCT: initiation of hormone corticosteroid therapy. DEXA: dual-energy X-ray absorptiometry

A 24-year-old male presented to our outpatient endocrinology clinic for evaluation of metabolic bone disease in the context of recurrent nephrolithiasis. His medical history was notable for his first episode of kidney stones at the age of six, identified as calcium oxalate in composition. This event prompted a referral to pediatric endocrinology. Although the specific rationale for imaging was not documented, a dual-energy X-ray absorptiometry (DEXA) scan was performed at that time and revealed significant osteoporosis with an L4 Z-score of -3.0 (Figure 1). Initial laboratory workup showed no abnormalities in serum calcium, phosphorus, parathyroid hormone (PTH), or vitamin D levels. Based on the DEXA findings, the patient was started on calcium and vitamin D supplementation. Over a five-year period of consistent therapy and lifestyle modifications - including weight-bearing exercise and dietary adjustments - his bone mineral density significantly improved, with a repeat L4 Z-score of -1.4 (Figure 1). After this period of improvement, the patient discontinued follow-up and treatment during adolescence.

In May 2022, at the age of 21, he experienced a recurrence of nephrolithiasis, which he associated with the recent initiation of regular physical exercise. Ultrasonography and urologic evaluation confirmed a 6 mm ureteral stone with moderate dilation of the left renal pelvis and calyces. Lithotripsy was performed, and stone analysis demonstrated a mixed composition of calcium oxalate and phosphate. Given the recurrence of urolithiasis and his history of childhood-onset osteoporosis, he was referred back to endocrinology for comprehensive evaluation.

During the endocrinologic consultation, the patient reported mild but persistent musculoskeletal complaints, including a sensation of joint clicking and intermittent pain localized to the thoracic and lumbar spine. He denied any recent fractures, trauma, or systemic symptoms such as weight loss, fever, or fatigue. His BMI was 19.75 kg/m² (height: 180 cm; weight: 64 kg), and physical examination revealed mild joint hypermobility and crepitus in the knees, without overt deformities or signs of inflammation.

Repeat DEXA scans revealed advanced osteoporosis, particularly in the forearm, with a T-score of -4.4 (Figure 1) on the left and -3.6 on the right. This prompted a renewed metabolic workup. Laboratory testing revealed the following key findings: slightly low corrected serum calcium, normal PTH levels, elevated 24-hour urinary calcium excretion, and low vitamin D levels. Of note, the 25-hydroxy-vitamin D level was 21.8 ng/mL prior to re-initiation of supplementation. Serum phosphate, magnesium, renal function, thyroid function (thyroid-stimulating hormone and free thyroxine), and liver enzymes were all within normal limits (Table 1).

**Table 1:** Comprehensive laboratory panel at initial endocrinology evaluation Comprehensive laboratory evaluation of the patient upon initial presentation to our department, following DEXA results that revealed advanced osteoporosis. The panel includes relevant hormonal, metabolic, and biochemical parameters guiding further diagnostic workup. Bold values indicate significant findings. ALP: alkaline phosphatase; ALT: alanine aminotransferase; AST: aspartate aminotransferase; DEXA: dual-energy X-ray absorptiometry; FSH: follicle-stimulating hormone; FT: free testosterone; FT4: free thyroxine; GGT: gamma-glutamyl transferase; HDL: high-density lipoprotein; LH: luteinizing hormone; LDL: low-density lipoprotein; PTH: parathyroid hormone; SHBG: sex hormone-binding globulin; TSH: thyroid-stimulating hormone

Test	Result	Reference range
PTH	33 ng/L	11-43 ng/L
Albumin	52 g/L	35-52 g/L
Total calcium	2.42 mmol/L	2.15-2.50 mmol/L
Corrected calcium	2.12 mmol/L	2.15-2.50 mmol/L
Phosphorus	1.39 mmol/L	0.81-1.45 mmol/L
25-OH vitamin D	21.8 ng/mL	30-100 ng/mL
24-hour urine calcium	12.3 mmol/24h	2.5-7.5 mmol/24h
TSH	2.4 uIU/mL	0.27-4.2 uIU/mL
FT4	14.2 pmol/L	12-22 pmol/L
Glucose	91 mg/dL	70-110 mg/dL
Creatinine	1.1 mg/dL	0.6-1.2 mg/dL
ALT	20 U/L	≤40 U/L
AST	18 U/L	≤40 U/L
GGT	10 U/L	≤60 U/L
ALP	41 U/L	40-130 U/L
Total bilirubin	0.7 mg/dL	≤1.2 mg/dL
Direct bilirubin	0.2 mg/dL	<0.3 mg/dL
Total cholesterol	162 mg/dL	<200 mg/dL
HDL	44 mg/dL	≥40 mg/dL
LDL	120 mg/dL	<130 mg/dL
Triglycerides	85 mg/dL	<150 mg/dL
Ferritin	75 µg/L	30-400 µg/L
Iron	1511 µg/L	330-1930 µg/L
Vitamin B12	287 pmol/L	145-569 pmol/L
Prolactin	34.51 ng/mL	4.8-23.3 ng/mL
LH	7.08 mU/mL	1.70-8.60 mU/mL
FSH	6.63 mU/mL	1.50-12.40 mU/mL
Total testosterone	7.50 µg/L	2.49-8.36 µg/L
SHBG	30 nmol/L	18-54 nmol/L
Free testosterone	0.179 µg/L	0.057-0.178 µg/L

Parathyroid scintigraphy (99mTc-sestamibi) showed no evidence of parathyroid adenomas or hyperplasia. Bone turnover markers, including alkaline phosphatase and total protein, were not elevated, further excluding high bone turnover states. Spine and brain MRI were also unremarkable, ruling out structural causes for musculoskeletal complaints and headaches, respectively. Given the combination of hypercalciuria and osteoporosis in the absence of hyperparathyroidism, we considered a diagnosis of idiopathic hypercalciuria.

The therapeutic approach included re-initiation of calcium (1000 mg/day) and vitamin D supplementation (50,000 IU weekly), aiming to support bone health and normalize serum vitamin D levels. To address the persistent hypercalciuria and mitigate the risk of stone recurrence, hydrochlorothiazide (25 mg daily) was added to the regimen. This resulted in a notable reduction in urinary calcium excretion, which fell to 7.78 mmol/24h. Simultaneously, serum calcium normalized (corrected calcium 2.3 mmol/L), and vitamin D levels stabilized within the desired range (35-45 ng/mL).

However, after a few weeks on hydrochlorothiazide, the patient reported new-onset erectile dysfunction, which he attributed to the thiazide diuretic. After a shared decision-making discussion and considering the impact on quality of life, hydrochlorothiazide was discontinued. Other causes of erectile dysfunction, including testosterone deficiency or hyperprolactinemia, were excluded based on normal hormonal profiles. Laboratory results showed total testosterone at 7.5 μg/L (normal range: 2.7-10.7 ng/mL), free testosterone within the normal calculated range, LH and FSH within reference limits, and prolactin levels fluctuating between 16 and 34 ng/mL.

The case was reviewed in a multidisciplinary setting including endocrinology, urology, and nephrology. Additionally, we contacted an osteology specialist in Germany for an external consultation. The specialist reviewed the clinical history and laboratory data and approved all diagnostic and therapeutic steps taken, without suggesting any modifications to the treatment plan. It was concluded that the patient most likely has IJO, and the clinical goal would be to maintain calcium and vitamin D repletion while exploring alternative agents to reduce urinary calcium without impacting sexual function.

His family history was notable for a paternal aunt diagnosed with premenopausal osteoporosis and a father who suffered a fatal cerebral aneurysm at age 35. He had no history of smoking in the last year and had no alcohol or substance use.

To date, the patient remains under close endocrinological follow-up. His most recent DEXA scan demonstrated improvement in osteopenic levels (T-score: -1.5, left forearm), and he is currently asymptomatic. We continue to monitor urinary calcium levels and bone density at regular intervals.

## Discussion

IJO is a diagnosis of exclusion and remains among the rarest primary bone disorders in pediatrics. Its clinical course is variable and poorly understood, with some patients entering remission during puberty, while others experience chronic symptoms persisting into adulthood. Although traditionally defined by its prepubertal onset, adult presentations have been reported, particularly when initial disease is undiagnosed or relapses after apparent remission [7,8].

Our case contributes to the growing recognition that IJO may exhibit a relapsing or smoldering course, especially in patients with partial response to early therapy. The patient’s early-onset osteoporosis with significant improvement under calcium and vitamin D supplementation, followed by recrudescence of symptoms in early adulthood, aligns with such a relapsing phenotype. While IJO is typically diagnosed before puberty, adult-onset or relapsing forms have been described, often raising questions about overlap with undiagnosed genetic bone disorders or other metabolic etiologies.

From a diagnostic perspective, IJO remains a diagnosis of exclusion. In this case, extensive metabolic and genetic workups ruled out other causes, such as osteogenesis imperfecta, X-linked hypophosphatemia, Cushing’s syndrome, chronic renal disease, and nutritional deficiencies. Although genetic testing was not performed due to limited availability, emerging research points to pathogenic variants in genes such as WNT1, LRP5, COL1A1, and COL1A2, which may account for cases historically labeled as idiopathic [9,10]. As access to next-generation sequencing improves, genetic screening may play an increasingly important role in refining the diagnosis and subclassification of pediatric and young adult osteoporosis.

One notable feature in this patient was the coexistence of idiopathic hypercalciuria, an entity frequently linked to increased fracture risk and reduced bone mass in both children and adults [11,12]. The mechanistic association may involve excessive urinary calcium loss contributing to negative calcium balance and impaired bone mineralization. Emerging evidence from pediatric cohorts indicates that idiopathic hypercalciuria may independently predict lower lumbar spine Z-scores, even in the absence of systemic illness or steroid exposure [13]. While the relationship between hypercalciuria and IJO remains unclear, its presence in this case may reflect either a comorbid trait exacerbating bone fragility or an intrinsic component of the disease process itself.

Management strategies for IJO remain largely empirical due to the paucity of randomized trials. Current consensus recommends lifestyle optimization, calcium and vitamin D repletion, and weight-bearing exercise as first-line interventions [14]. Bisphosphonate therapy, while increasingly used in severe pediatric osteoporosis, remains controversial in IJO due to limited long-term safety data and unclear efficacy in this specific subgroup [15].

Our patient showed a reduction in urinary calcium levels with hydrochlorothiazide therapy, a thiazide diuretic known to reduce calciuria by enhancing renal calcium reabsorption in the distal tubule [16]. However, the development of erectile dysfunction, although an uncommon but recognized side effect, prompted discontinuation. In such cases, alternative strategies may include dietary sodium restriction, reduced animal protein intake, or use of potassium citrate, which can reduce stone formation risk and mitigate calciuria by alkalinizing the urine and inhibiting calcium crystallization.

The interplay between bone fragility and calcium handling disorders like hypercalciuria suggests a possible shared pathophysiologic substrate. Longitudinal studies have shown that persistent hypercalciuria may predict poorer bone outcomes and reduced bone mineral accrual into adulthood [13,16]. It remains unclear whether hypercalciuria in this case is a manifestation of IJO, a parallel metabolic abnormality, or a modifying factor that influences disease severity.

Interdisciplinary collaboration played a pivotal role in this case. A multidisciplinary team, including endocrinology, urology, and nephrology, supplemented by external consultation with an osteology specialist, ensured a robust evaluation of potential diagnostic and therapeutic strategies. This model of care is especially critical in rare diseases like IJO, where no standardized clinical pathways exist.

This case underscores the importance of lifelong vigilance in patients with childhood-onset osteoporosis, particularly when apparent remission may mask ongoing metabolic vulnerability. The coexistence of idiopathic hypercalciuria may serve as a biomarker of relapse risk or treatment resistance, warranting closer surveillance, individualized therapy, and consideration of genetic investigation when available.

## Conclusions

IJO is a rare condition that can persist or recur beyond childhood and should be considered in the differential diagnosis of unexplained osteoporosis in young adults, even in the absence of a known fracture history. This case highlights the importance of maintaining clinical suspicion for IJO when other secondary causes are excluded.

The coexisting idiopathic hypercalciuria likely exacerbated ongoing bone loss, underscoring the relevance of evaluating calcium metabolism disorders as potential contributing factors. Effective management in this case required calcium and vitamin D supplementation, lifestyle modifications, and regular monitoring. The discontinuation of hydrochlorothiazide due to adverse effects illustrates the importance of personalized therapeutic approaches. Long-term follow-up is essential to monitor disease progression, treatment response, and potential relapse. This case reinforces the need for heightened awareness, individualized care plans, and further research into both IJO and its metabolic associations.
